# Changes of Resurgent Na^+^ Currents in the Na_v_1.4 Channel Resulting from an *SCN4A* Mutation Contributing to Sodium Channel Myotonia

**DOI:** 10.3390/ijms21072593

**Published:** 2020-04-08

**Authors:** Chiung-Wei Huang, Hsing-Jung Lai, Pi-Chen Lin, Ming-Jen Lee

**Affiliations:** 1 Institute of Physiology, Kaohsiung Medical University, Kaohsiung 80708, Taiwan; g10054b@ms51.hinet.net; 2 Department of Neurology, National Taiwan University Hospital, Taipei 10051, Taiwan; 3 Department of Neurology, National Taiwan University Hospital Jinshan Branch, New Taipei City 20844, Taiwan; i5492111@gmail.com; 4 Division of Endocrinology and Metabolism, Department of Internal Medicine, Kaohsiung Medical University Hospital, Kaohsiung Medical University, Kaohsiung 80708, Taiwan; pichli@kmu.edu.tw; 5 Department of Neurology, National Taiwan University Hospital Yunlin Branch, Yunlin 10051, Taiwan

**Keywords:** myotonia congenita, *SCN4A* mutation, sodium channel, Nav1.4, resurgent current

## Abstract

Myotonia congenita (MC) is a rare disorder characterized by stiffness and weakness of the limb and trunk muscles. Mutations in the *SCN4A* gene encoding the alpha-subunit of the voltage-gated sodium channel Na_v_1.4 have been reported to be responsible for sodium channel myotonia (SCM). The Na_v_1.4 channel is expressed in skeletal muscles, and its related channelopathies affect skeletal muscle excitability, which can manifest as SCM, paramyotonia and periodic paralysis. In this study, the missense mutation p.V445M was identified in two individual families with MC. To determine the functional consequences of having a mutated Na_v_1.4 channel, whole-cell patch-clamp recording of transfected Chinese hamster ovary cells was performed. Evaluation of the transient Na^+^ current found that a hyperpolarizing shift occurs at both the activation and inactivation curves with an increase of the window currents in the mutant channels. The Na_v_1.4 channel’s co-expression with the Na_v_β4 peptide can generate resurgent Na^+^ currents at repolarization following a depolarization. The magnitude of the resurgent currents is higher in the mutant than in the wild-type (WT) channel. Although the decay kinetics are comparable between the mutant and WT channels, the time to the peak of resurgent Na^+^ currents in the mutant channel is significantly protracted compared with that in the WT channel. These findings suggest that the p.V445M mutation in the Na_v_1.4 channel results in an increase of both sustained and resurgent Na^+^ currents, which may contribute to hyperexcitability with repetitive firing and is likely to facilitate recurrent myotonia in SCM patients.

## 1. Introduction

The human *SCN4A* gene encodes the alpha-subunit of the voltage-gated sodium channel Na_v_1.4, which is the pore-forming subunit. The Na_v_1.4 channel is expressed in skeletal muscles, and its related channelopathies affect skeletal muscle excitability [1,2,3]. There are two prevalent clinical symptoms: (1) muscle stiffness with hypertonia (myotonia) episodes (non-dystrophic myotonia, NDM); and (2) muscle weakness leading to recurrent paralysis episodes (periodic paralysis, PP). According to clinical and electromyographical (EMG) features, there are at least two disease entities for NDM: sodium channel myotonia (SCM) and paramyotonia congenita (PMC). The clinical features of dominant SCM are not significantly cold-sensitive and do not significantly decrease the compound motor action potential in an EMG test. SCM can manifest as paramyotonia, potassium-aggravated myotonia, myotonia permanens, acetazolamide-responsive myotonia, painful myotonia and severe neonatal episodic laryngospasm [4]. Patients with SCM may sometimes present with exercise-induced, delayed-onset myotonia or acetazolamide-responsive myotonia [5]. An EMG examination will show a progressive decrease of compound motor action potential in a post-repetitive short effort test. Furthermore, PP caused by Na_v_1.4 channelopathy can be divided into hypokalemic PP (hypoPP) and hyperkalemic PP (hyperPP), which are associated with the blood potassium level. Generally, overlap, borderline or mixed clinical features between NDM and PP can be identified in patients with a Na_v_1.4 mutation [6,7,8,9]. The age of onset is from early to late childhood. The prevalence of Na_v_1.4 channelopathies involving skeletal muscle has been estimated to be 0.4~1.4 : 100,000 [5]. As of today, more than seventy mutations in the *SCN4A* gene have been identified as pathogenic, and twelve are likely pathogenic [10]. Most *SCN4A* mutations are transmitted dominantly. The penetrance of the mutations among pedigrees is variable. Thus, the variable genotypes and phenotypes render it difficult to identify a correlation among Na_v_1.4 channelopathies.

Changes of the electrophysiological features with a Na_v_1.4 mutation in functional expression models have been investigated for two decades. The repertoire of biophysical defects of mutant channel behavior that predisposes the affected muscle affect both activation and inactivation gating mechanisms [10]. Basically, a Na_v_1.4 gain of function mutation results in myotonia, hyperPP and hypoPP; however, some defects predispose to myotonia, while others increase susceptibility to PP. Previous studies on Na_v_1.4 mutations in SCM patients have demonstrated that the kinetics of channel inactivation are altered with a slower rate of onset and, sometimes, with faster recovery as well [11,12]. Nevertheless, the most characteristic phenotype, myotonia, as shown in EMG, demonstrates repetitive firing of action potentials. Considering the repetitive discharge, clinical phenotypes such as ataxia in *Scn8a*-deficient mice, and recurrent attacks of erythromelalgia in patients with an *SCN9A* mutation have been proposed to be ascribed to resurgent Na^+^ currents elicited during the repolarization stage [13,14]. The genesis of resurgent current occurred at repolarization stage with reopening of the voltage-gated sodium channel. It has been proposed that there is competition between the Na_v_β4 peptides and the inactivating molecules to block the reopened channel [14,15,16,17]. Nevertheless, how the mutations in voltage-gated sodium channel facilitate the occurrence of resurgent current remains elusive.

As of today, the resurgent Na^+^ currents found in the Na_v_1.4 channel and the impact of a Na_v_1.4 mutation on SCM have yet to be characterized, aside from a single study on PMC in dorsal root ganglion (DRG) neurons [18]. Studying a known mutation in the Na_v_1.4 channel in two individual families with SCM, our research aims to determine the changes of the resurgent Na^+^ current caused by the mutation in the Na_v_1.4 channel and correlate the phenotype with its aberrant electrophysiological features.

## 2. Results

### 2.1. Genetic Findings from Two Families with Myotonia Congenita (MC)

**Figure 1:**
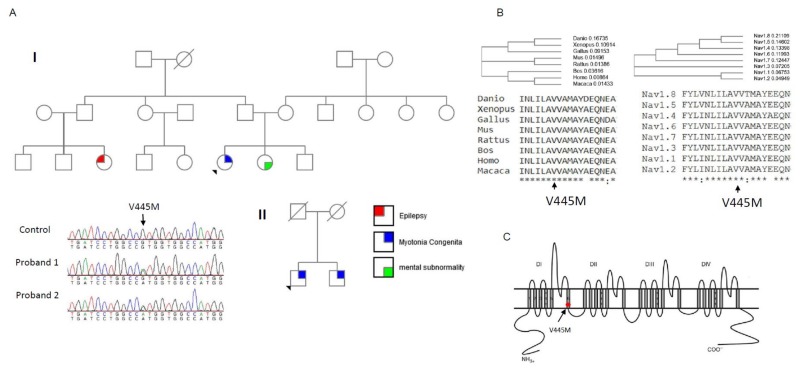
Sequence chromatography of the mutation and multiple sequence alignments for Na_v_1.4 channels. (**A**) The pedigrees from two individual families (Family I and II) with the index cases are shown in the upper and lower panels, respectively. Both index cases suffered from muscle myotonia with muscle hypertrophy. In Family I, the sister of the index case suffered from mental subnormality and one of her relative had seizure history. Both the index case and his brother from Family II suffered from myotonic myopathy. The sequence chromatography (left side of lower panel) shows a variant, c.G1333A at exon 9 of the *SCN4A* gene, which causes a p.V445M mutation. Both probands have the sequence variant. The variant is not found in the relatives of the index case of Family I. In Family II, both the proband and his brother share the same sequence variant. (**B**) The amino acid V445 is highly conserved among species during evolution and in the protein family of voltage-gated sodium channels. (**C**) The topology of the Na_v_1.4 channel shows that the mutation is located at the sixth transmembrane segment of Domain I (DI/S6).

Two probands from two individual Taiwanese–Chinese families were selected. They had developed stiffness with difficulty relaxing their limb and truncal muscles. No periodic weakness occurred, and their muscle bulk was conspicuously large as compared with the age-matched controls. According to the family pedigree (Family I, upper panel of Figure 1A), the index case developed NDM, his sister had mental subnormality, and one of his cousins had a history of epilepsy. The nuclear family (Family II, lower panel of Figure 1A) with two affected brothers developed similar features of NDM. The candidate gene screening for a *CLCN1* mutation did not find any sequence variant; however, further sequencing of *SCN4A* gene identified a missense variant, c.1333G>A, that results in the p.V445M change in both index cases. The DNA from the parents of the proband in Family I was not available. Although the parents of Family II were dead, both the index case and his brother harbored the mutation. The amino acid Val445 was found to be not only conserved across the species in evolution but also across the alpha-subunits of the human voltage-gated sodium channel protein family (Figure 1B). Topologically, the p.V445M mutation is located at the sixth transmembrane α–helix of the first domain (DI/S6, Figure 1C). The mutation has been reported to be pathogenic in previous studies [19,20].

### 2.2. A Smaller Sustained Na^+^ Current in the WT Channel than in the p.V445M Mutant Channel

**Figure 2:**
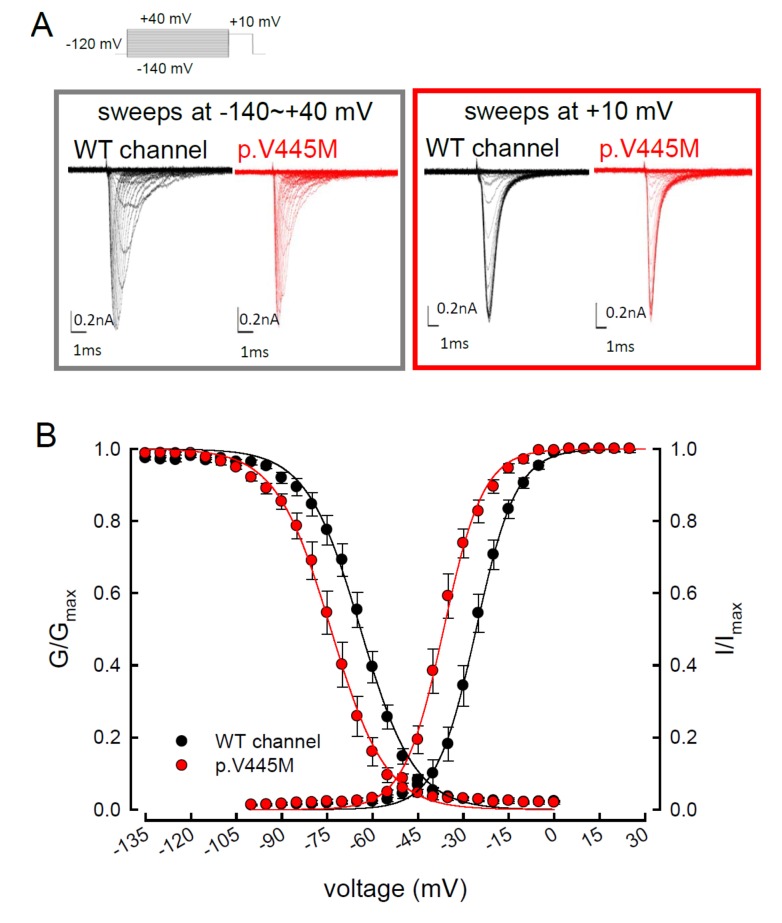
Activation and inactivation curves of wild-type (WT) and p.V445M mutant channels in the CHO-K1 cells. (**A**) Sample sweeps of the activation (left panel) and inactivation (right panel) curves of the WT and p.V445M mutant channels with 0.1 mM Na_v_β4 peptide. For the current-voltage (I-V) plot, each patch cell configuration was held at −120 mV, and step test pulses were from −140 to +40 mV for 100 ms in each 5 mV escalation and plotted against test pulse voltage where each with 5 potential step. For the inactivation curve of Na^+^ currents, the maximal currents at +10 mV were documented after 100 ms prepulses at various voltages. (**B**) The activation and fast steady-state inactivation curves of the WT and p.V445M mutant channels are fitted with a Boltzmann function according to the following formula: 1 / [1 + exp((V_h_ − V) / *k*)], where V is the membrane potential, V_h_ and *k* are −25.79 ± 1.5 mV and 6.67 ± 0.43 for the activation curve and −63.99 ± 2.17 mV and −8.93 ± 0.33 for the inactivation curve in the WT Na_v_1.4 channel; −36.53 ± 1.4 mV and 6.61 ± 0.23 for the activation curve and −73.81 ± 1.2 mV and −9.11 ± 0.21 for the inactivation curve in the p.V445M mutant channel, respectively (*n* = 9 for each measurement).

**Figure 3:**
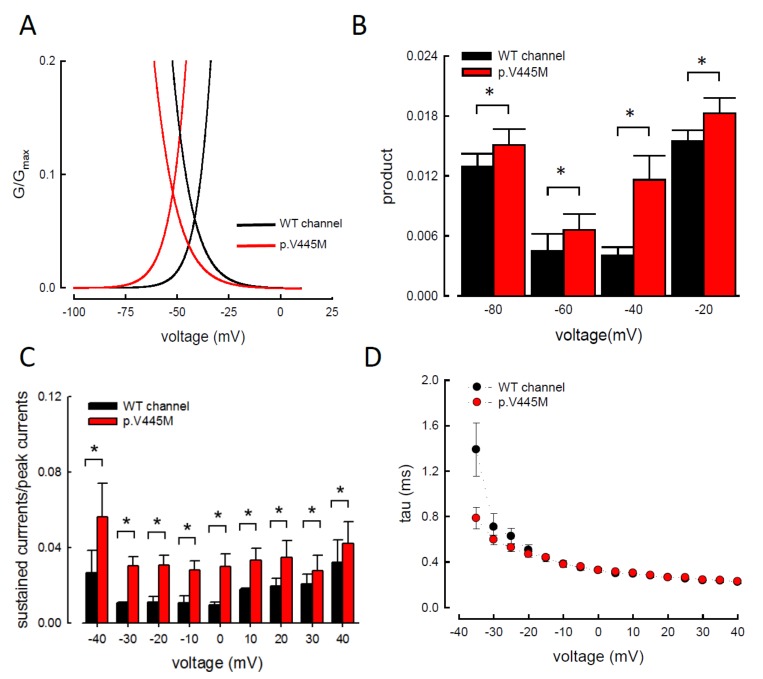
Larger window currents in the p.V445M mutant channel in the Chinese hamster ovary (CHO-K1) cells (**A**) An enlarger view of Figure 2B between −100 and +5 mV. (**B**) The product of G/G_max_ and I/I_max_ at each voltage from Figure 1B is plotted against the different voltages in the WT and p.V445M mutant channels (*, *p* < 0.05 compared to the WT). (**C**) The ratio between the sustained (the average currents between 90 and 95 ms of the pulse) and peak (maximal) transient currents at +40 to −40 mV (sample sweeps in Supplementary data Figure S2) is significantly smaller in the WT channels in comparison with the p.V445M mutant channels (*, *p*<0.05 between the WT and p.V445M mutant channel). (**D**) Fast-inactivation kinetics of WT and p.V445M mutant channels. The inactivation time constants were obtained by fitting the decay phase of transient sodium currents (Na_T_) shown in Figure 2A with a mono-exponential function. Data are expressed as means ± SEM.

We compared the electrophysiological characteristics of the basic gating control of the WT and p.V445M mutant Na_v_1.4 channels. Figure 2A shows the sweeps after the depolarization via either an escalating (from −140 to +40 mV, left panel of Figure 2A) or constant (+10 mV, right panel of Figure 2A) depolarizing current. The activation and inactivation curves of the WT and p.V445M mutant channels were fitted with the following Boltzmann function: 1 / [1 + exp((V_h_ − V) / *k*)], where V is the membrane potential and *k* is the slope factor. From the equation, V_h_ and *k* were −25.79 ± 1.5 mV and 6.67 ± 0.43 for the activation curve and −63.99 ± 2.17 mV and −8.93 ± 0.33 for the fast steady-state inactivation curve in the WT channel, respectively and −36.53 ± 1.4 mV and 6.61 ± 0.23 for the activation curve and −73.81 ± 1.2 mV and −9.11 ± 0.21 for the inactivation curve in the p.V445M mutant channel, respectively (*n* = 9; *p* < 0.05; Figure 2B). Both the activation and fast steady-state inactivation curves in the p.V445M mutant channel were hyperpolarization-shifted compared with those in the WT channel. The slopes of both gating curves of the Na_v_1.4 channels remained grossly unchanged (Figure 2B). We further analyzed the window currents, which were defined by the area under the activation and fast steady-state of the inactivation curves. The window currents obviously increased in the mutant channel as compared with the WT channel (Figure 3A), although hyperpolarization shifting was observed at both the activation and inactivation curves. Figure 3B shows the products of the activation and inactivation curves, which indicate that the window currents or sustained Na^+^ currents were significantly larger in the mutant channels than in the WT channels at different membrane potentials (i.e., −20, −40, −60 and −80 mV). Moreover, the ratio between the sustained and peak currents in the p.V445M mutant channel was significantly larger than that of the WT channel (Figure 3C). The increase of a sustained Na^+^ current associated with a large window current may partially account for the phenotype of hyperexcitability. Theoretically, increased sustained Na^+^ currents could imply a decreased inactivation rate and/or increase of a reverse rate. These findings suggest that the p.V445M mutation plays a role as a gating modifier of the Na_v_1.4 channel. To investigate whether the kinetics of fast-inactivation were changes by p.V445M mutant channel, we measure the time constants of fast-inactivation (Figure 3D). The inactivation time constants were measured by fitting the decaying phases of transient sodium currents (Na_T_) from 80% of maximal to the end with a mono-exponential function. The inactivation rate of p.V445M was faster than that of WT channel at -35 mV but it was not significantly different at more depolarized potentials.

### 2.3. The p.V445M Mutant Channel Increases Resurgent Na^+^ Currents

**Figure 4:**
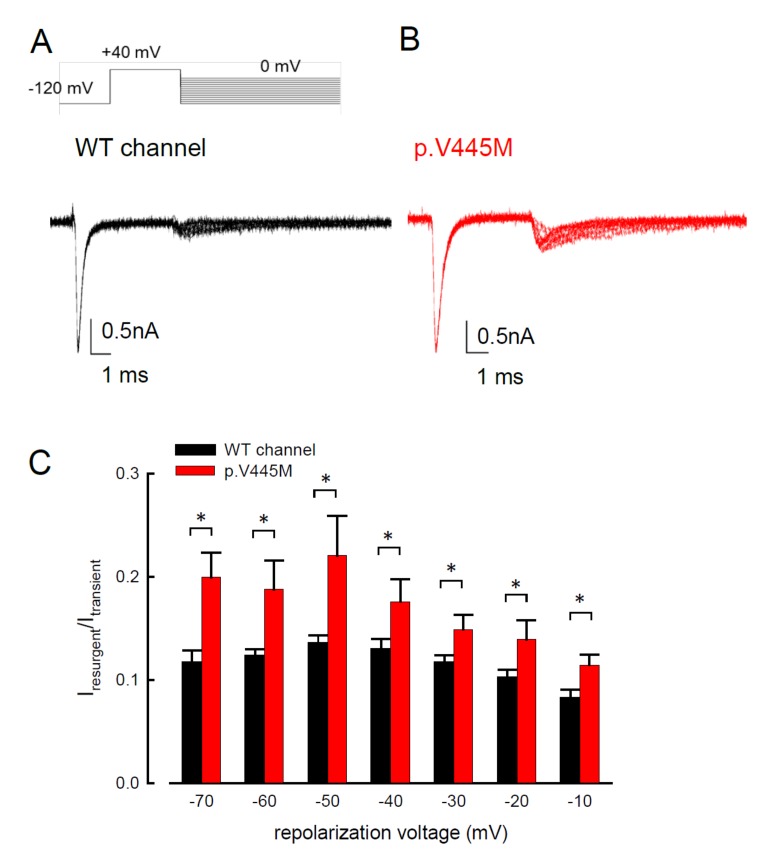
Smaller resurgent Na^+^ currents in the WT channel than that in the p.V445M mutant channel in the CHO-K1 cells (**A**) The protocol to generate resurgent Na^+^ currents is as follows: the patched cells were held at −120 mV, and the resurgent Na^+^ currents of the WT channel were evoked by pulses following a depolarization prepulse of +40 mV for ~10 ms in the presence the Na_v_β4 peptide (upper panel). Sample sweeps for the WT channel show the occurrence of resurgent Na^+^ currents at ~4 ms post repolarization in the presence of 100 μM Na_v_β4 peptide (lower panel). (**B**) Sample sweeps for the p.V445M mutant channel in the presence 0.1-mM Na_v_β4 peptide following the same protocol as in part **A.** (**C**) The ratio between resurgent and peak transient currents is significantly smaller in the WT than in the p.V445M mutant channel between −70 and −10 mV. (*, *p* < 0.05).

Resurgent Na^+^ currents can be observed in voltage-gated Na^+^ channels containing a Na_v_β4 peptide at the repolarization stage [21]. Figure 4 shows the resurgent Na^+^ currents elicited at the repolarization (0 to −100 mV) following a prepulse depolarization at +40 mV in the p.V445M mutant and WT Na_v_1.4 channels. The sweeps show that the magnitude of the resurgent Na^+^ currents in the p.V445M mutant channel was larger (Figure 4B) compared with that of the WT channel (Figure 4A). Upon evaluating the ratio between the resurgent and transient currents, the mutant channel had a significantly high ratio at the repolarizing voltages, from −70 mV up to −10 mV (Figure 4C). These results demonstrate that the p.V445M mutation located at Domain I/segment VI (DI/S6) may play a role in the genesis of resurgent Na^+^ currents that is mediated by the inter-transformation between inactive and open (activation) states of the channel (refer to Discussion). The changes in the gating conformation caused by p.V445M mutant channels facilitate repetitive firing, which, in turn, can induce a drastic increase in muscular stiffness and clinical symptoms of SCM. We also evaluated whether the activation or inactivation curves can be modified by the peptide of Na_v_β4, which we added during the genesis of resurgent current. Both activation and inactivation curves of the WT Na_v_1.4 channel are evidently hyperpolarized shift on the membrane potential axis with the presence of 0.1 mM Na_v_β4 peptides in comparison those without the peptides (Supplementary data Figure S1). However, the slope of the curves is unchanged. The window current remains unchanged either in the presence or absence of Na_v_β4 peptide.

### 2.4. Evidence of Two Open States for Transient and Resurgent Na^+^ Currents in the WT Na_v_1.4 Channel

**Figure 5:**
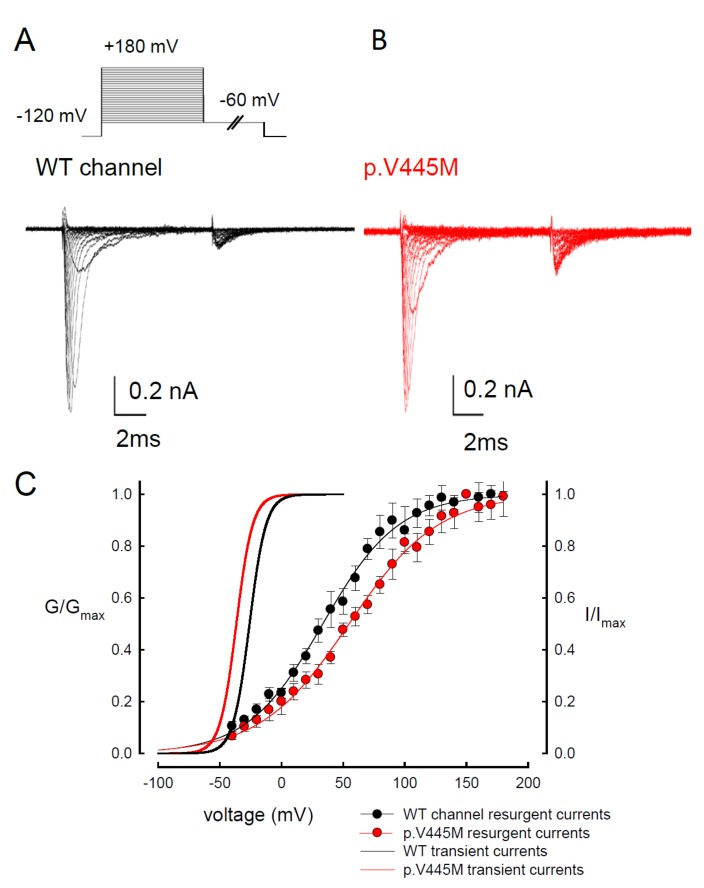
Activation curve of resurgent Na^+^ currents in the CHO-K1 cells (**A**) The protocol to generate resurgent Na^+^ currents are as follows: the cell was held at −120 mV for ~30 ms, and then stepped to various depolarization between −60 and +180 mV for 5 ms in 10 mV increments. Resurgent Na^+^ currents were then evoked by a pulse of −60 mV for 150 ms (upper panel). Sample sweeps were obtained in the presence of 0.1 mM Na_v_β4 peptide for the WT channel (lower panel). (**B**) Sample sweeps for the p.V445M mutant channel in the presence 0.1 mM Na_v_β4 peptide. (**C**) The activation curves of transient and resurgent Na^+^ currents for the WT and p.V445M mutant channels are replotted for comparison. The activation curve of resurgent Na^+^ currents of the WT channel (the block point fitting line) for each cell are obtained by fittings with Boltzmann functions, and the cumulative results for V_h_ are +33.64 ± 1.7 mV and *k* are 30.45 ± 1.45 for ~10 ms prepulses, respectively (*n* = 8). The resurgent activation curve of the p.V445M mutant channel (the red-point-fitting line) for each cell are also obtained by fitting Boltzmann functions and the cumulative results for V_h_ and *k* are +54.04 ± 1.43 mV (*n* = 6) and 33.84 ± 1.12 (*n* = 6), respectively .

We observed the changes of resurgent Na^+^ currents with a trend of escalating depolarization prepulses between −60 and +180 mV. Depolarization via prepulses within 10 ms could produce a similar activation curve of resurgent Na^+^ currents in both the WT and p.V445M mutant channels (Figure 5A,B). The 10 ms of depolarization induced resurgent Na^+^ currents, indicating that an open (activation) state of the Na_v_1.4 channel can be obtained. The activation curves of the transient Na^+^ currents and those of the resurgent Na^+^ currents of the WT and mutant channels were replotted. The activation curve of the transient sodium current showed a hyperpolarizing shift in the mutant channel (Figure 5C), which is in line with our previous study (Figure 2B). However, the activation curve of the resurgent Na^+^ currents moved to a more depolarized membrane voltage range in the mutant channel compared with the WT channel (Figure 5C). After fitting with Boltzmann functions, the activation curves of the resurgent Na^+^ currents from the WT channel showed that the cumulative results were +33.64 ± 1.7 mV for V_h_ and 30.45 ± 1.45 for *k*, while for the p.V445M mutant channel, the results were 54.04 ± 1.43 mV for V_h_ (*n* = 6; *p* < 0.05) and 33.84 ± 1.12 for *k* (*n* = 6; *p* < 0.05) (Figure 5C). In both the WT and p.V445M mutant channels, the activation curve for the transient Na^+^ current was in a much lower range of membrane potential than that of the resurgent Na^+^ currents (Figure 5C). The slope of the activation curve of the resurgent Na^+^ current was much less steep. These findings suggest that there may be two open states (O_1_ and O_2_) for the channel during activation (refer to Discussion).

### 2.5. Changes of the Decay Rate and Time to Peak of Resurgent Na^+^ Currents in Mutant Channels

**Figure 6:**
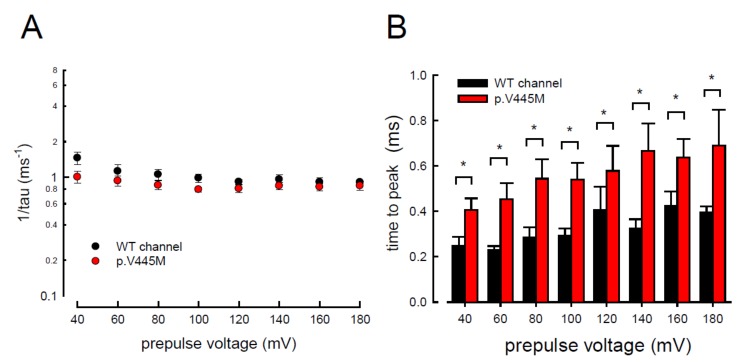
Accelerated decay of resurgent Na^+^ currents by hyperpolarization between the WT and p.V445M mutant channels in the CHO-K1 cells (**A**) The inverses of decay time constants (1/tau) of the resurgent Na^+^ currents are obtained at −60 mV after the depolarizing prepulses between +40 and +180 mV in 20 mV increments for ~10 ms in the WT and p.V445M mutant channels (*n* = 8). (**B**) The time to peak of resurgent Na^+^ currents in the WT channel is always faster than that in the p.V445M mutant channel at the prepulse voltages tested (*n* = 8 *; *p* < 0.05).

**Figure 7:**
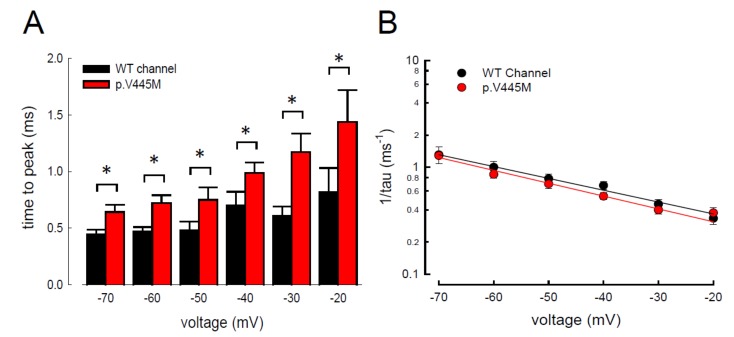
Slower time to peak of the resurgent Na^+^ currents in the p.V445M mutant channel in the CHO-K1 cells (**A**) The time to the resurgent current peak was measured with the same protocol in Figure 4 (*n* = 6). The time to the resurgent Na^+^ current peak is significantly shortened in the WT than in the p.V445M mutant channel between −20 and −70 mV (*; *p* < 0.05). (**B**) The reciprocal of the time constants (1/tau) for the decay phase of resurgent Na^+^ currents was plotted against the voltage in semi-logarithmic scales for the WT and p.V445M mutant channels (*n* = 8). The lines are linear regression fits of the form: 1/tau_(V)_ = 0.22 × exp(−0.64V/25) ms^−1^ for the WT channel and 0.18 × exp(−0.69V / 25) ms^−1^ for the p.V445M mutant channel, respectively.

In addition to measuring the magnitude of the resurgent Na^+^ currents, we also examined the kinetic changes in the resurgent Na^+^ currents induced by the p.V445M mutation. As shown in Figure 6A, the decay kinetics of the resurgent Na^+^ currents were not significantly changed in the p.V445M mutant channel within a wide range of depolarizing prepulses (from +40 to +180 mV). However, the time to peak of the resurgent Na^+^ currents during repolarization was significantly longer in the mutant channel than in the WT channel (Figure 6B). The changes of these parameters under a series of repolarizing potentials from −70 up to −20 mV were also evaluated. Compared with the WT channel, the time to peak was significantly slower in the mutant channel during repolarization (Figure 7A). Moreover, the decay kinetics (1/tau) of the p.V445M mutant channel matched those of the WT channel without an obvious change (1/tau_(V)_ = 0.22 × exp(−0.64V/25) ms^−1^ for the WT channel and 1/tau_(V)_ = 0.18 × exp(−0.69V/25) ms^−1^ for the p.V445M mutant channel; Figure 7B). These findings suggest that two different individual open (activation) states are responsible for the transient and resurgent Na^+^ currents. The decay rate from the open (activation) state of the resurgent Na^+^ currents in the p.V445M mutant channel was the same as that observed in the WT channel. However, compared with those in the WT channel, the resurgent Na^+^ currents in the p.V445M mutant channel were conspicuously slow, indicating that the mutation results in a protracted period of open state of the Na_v_1.4 channel, either after strong depolarizing prepulses or under different repolarization potentials.

## 3. Discussion

In summary, we identified the missense mutation p.V445M of the *SCN4A* gene from two patients with SCM who were recruited from two individual families. To determine the electrophysiological function of this mutation, a transfected CHO-K1 cell model was employed for the whole-cell patch-clamp recording. Evaluation of the transient Na^+^ current showed a hyperpolarizing shift in both the activation and inactivation curves of the mutant channel as compared with the WT channel. The window and sustained Na^+^ currents of the mutant channel were larger than those of the WT. Upon transfection of both Na_v_1.4 and a Na_v_β4 peptide, resurgent Na^+^ currents could be obtained 3~5 ms after a prepulse of depolarization. Compared with that of the WT channel, the magnitude of the resurgent Na^+^ currents were conspicuously larger in the mutant channel. Moreover, the time to peak of the resurgent Na^+^ currents in the mutant channel was slower than that of the WT channel, although no difference was found between them in the decay kinetics.

Myotonia results from an involuntary muscle contraction that persists for several seconds after the cessation of voluntary effect [22]. The hyperexcitability of muscles with a burst of muscle action potentials leads to an after-contraction that can last for several seconds. The spontaneous, painless discharges show a waxing and waning pattern upon EMG study. A sustained burst of discharges can be elicited by a brief voluntary contraction, and the firing frequency is usually 20~80 Hz, which means the interval of the depolarizing potentials is between 12.5 and 50 ms [23]. The repetitive, high-frequency discharges in myotonia suggest that resurgent Na^+^ currents may be a critical factor involving the phenotype. Channels are usually refractory to be activated after inactivation until the membrane potential has been sufficiently hyperpolarized. Nevertheless, channels can reopen, allowing a surge of inward current during repolarization, i.e., resurgent Na^+^ currents. Raman and Bean first described resurgent Na^+^ currents in cerebellar Purkinje neurons [16]. In the cerebellum, the resurgent Na^+^ currents following relief of an ultra-fast open-channel block by an endogenous blocking particle, probably the C-terminal portion of the Na_v_β4 subunit [24], may contribute to high-frequency firing [16,25]. The Na_v_1.6 channel is the major carrier of resurgent Na^+^ currents in cerebellar [26] and DRG [27] neurons. In the whole-cell configuration of transfected CHO-K1 cells expressing the Na_v_1.7 channel, resurgent Na^+^ currents after a long depolarizing prepulse have also been identified [13]. Resurgent Na^+^ currents from the mutant Na_v_1.7 channel may play an important role in the episodic attacks of severe neuropathic pain found in both erythromelalgia and paroxysmal extreme pain disorders [28]. Enhancement of resurgent Na^+^ currents has even been proposed to be highly correlated with the severity of neuropathic pain [28]. In the present study, a hyperpolarizing shift was found in both activation and inactivation curves between the mutant and WT channels. Although the window currents increased in the mutant channel, the hyperexcitability with repetitive recurrent discharges could not be fully explained by the changes in the transient Na^+^ current alone. Here, we described the resurgent Na^+^ currents in the Na_v_1.4 channel. Compared with the WT channel, the larger magnitude and slowing of the time to peak of the resurgent Na^+^ currents in the mutant channel may be one of the crucial factors of the functional disturbances of SCM. It has been proposed that impaired inactivation is a major determinant of resurgent Na^+^ currents in Na_v_1.7 channels [28]. Future research should investigate whether mediation of the inactivation process governs the functional consequences resulting from a mutation in the Na_v_1.4 channel, although prolonged recovery of slow inactivation has been observed in the p.V445M mutant channel [20].

In our study, resurgent currents of Nav1.4 channel are discernible only in the presence of the Na_v_β4 peptides (Figure S1). According to the prevailing model, this is ascribable to the competition between the Na_v_β4 peptides and the inactivating peptide for the open channel pore [14,15,16,17]. However, quite other biophysical characteristics, including (1) the concomitant increase of resurgent and sustained currents with the p.V445M mutant channels (Figure 3A–C and Figure 4), (2) the very much different activation curves for the transient and resurgent currents (Figure 5C) all strongly argue against that the Na_v_β4 peptide competes with the inactivating molecule for the same single open state, (3) the slower time to peak but unchanged decay kinetics of the resurgent currents with p.V445M mutant channels also contradicts that Na_v_β4 peptide competes with the inactivating molecule for the same single open state to generate the resurgent currents. We therefore propose that there may be two distinct open states (with two corresponding inactivated states) of the Na_v_1.4 channel (Figure 5), each responsible for the transient (O_1_) and resurgent (O_2_) currents, respectively. Significant occupancy of O_2_ is possible only in the presence of the Na_v_β4 peptides, which is thus chiefly a gating modifier inducing new gating conformations rather than a pore blocker competing with the inactivating peptides.

The activation curves of the resurgent Na^+^ currents in both the WT and p.V445M mutant channels markedly moved to a more positive voltage range, with a much less steep slope than those of the transient currents (Figure 5C). We proposed that there may be two open states of the channel and that the gating modification illustrates the electrophysiological changes of the genesis of resurgent Na^+^ currents [13]. This study showed that either after a depolarizing prepulse (Figure 6B) or under a variable extent of repolarization (Figure 7A), the time to peak of the resurgent Na^+^ currents in the mutant channel was extended significantly compared with that in the WT channel; however, the decay kinetics (1/tau) remained similar between the channels. The p.V445M mutation significantly accelerated the entry and protracted the duration of the new open (activation) state, leading to an increase in the magnitude of the resurgent Na^+^ currents. The reopening of the fast inactivated channels was probably accompanied by conformational changes of the domain I/S6 (DI/S6), where V445M is located.

**Figure 8:**
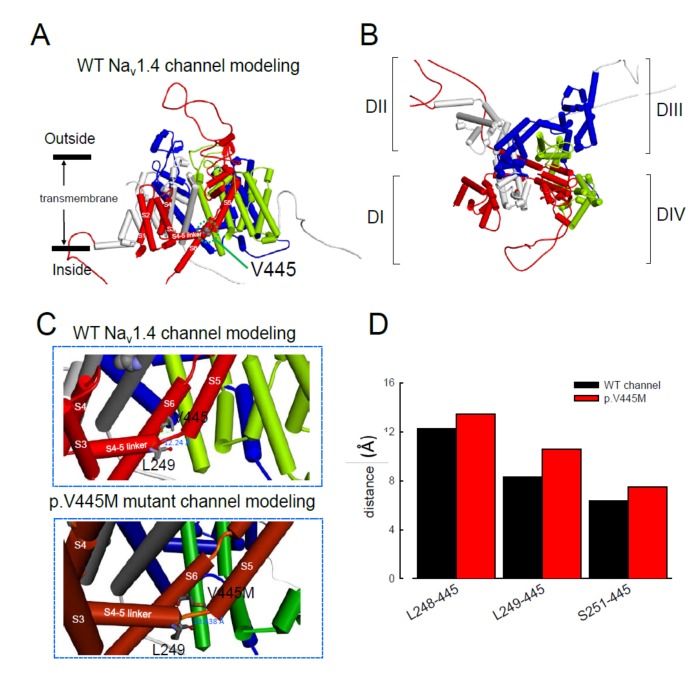
Homology modeling of the WT and mutant human Na_v_1.4 channels. (**A**) Side view of the homology model shows the transmembrane helixes of the four domains. The four domains I, II, III and IV are colored red, white, blue and green. The side chain of V445 in the S6 linker of Domain I (DI) is indicated in the CPK model. (**B**) A closer view of the WT Na_v_1.4 channel from the external side of the pore. (**C**) A diagram of Domain I of the homology model of the WT Na_v_1.4 and p.V445M mutant channels is shown in the schematic presentation. The side chains of V445 in the S6 segment and L248, L249 and S251 in the S4–5 linker of Domain I are shown with sticks of various colors. A closer view of the area is shown in the panel, demonstrating inter-residue distances of ~8.3196, ~12.24 and ~6.348 Å between V445 and L248, V445M and L249 and V445 and S251, respectively, in the WT Na_v_1.4 channel, while the distances between p.V445M and L248, p.V445M and S249 and p.V445M and S251 are ~7.54, ~13.438 and ~10.551 Å, respectively, in the p.V445M mutant channel. (**D**) The relative distances between the residue V445 and L248, L249 and S251 in the homology modeling is shown. Note the conspicuously decreased distances in all cases in the WT channel compared with those in the p.V445M mutant channel.

The computer homology model of the WT Na_v_1.4 channel evaluated the topological structure change caused by the p.V445M mutation (Figure 8A,B). The model estimated that the change of Val445 to Met may increase the distance between V445 and the residues at the S4–S5 linker (DI/S4-5 linker) (e.g., L248, L249 and S251; Figure 8). While the S4–S5 linker plays an imperative role in governing the activation/inactivation of resurgent Na^+^ currents [13], such a distance change could contribute to the attenuation of inactivation and uncoupling between activation and inactivation. In the previous study, genesis of resurgent Na^+^ currents in the Na_v_1.7 channel was found to be possibly due to the existence of two open states in activation [13]. Through modulating the transitional kinetics between these two states, the S4–S5 linker in Domain III seems to play an important role in activation–inactivation coupling. Mutations involving the linker and relevant nearby regions may, therefore, enhance the sustained and resurgent Na^+^ currents, leading to marked hyperexcitability and repetitive firing in the corresponding tissues. Herein, we postulated that the change of distance between V445 and the residues of the S4–S5 linker may be one of the critical factors for the coupling effect. Aberrations in modulation of the coupling facilitate the generation of resurgent Na^+^ currents and increase both the magnitude and time to peak, augmenting the repetitive firing. The mutation causes slowing of the time to peak of the resurgent Na^+^ currents elicited in the Na_v_1.4 channel, which increases the firing frequency of the muscle membrane. The action potential propagates to the T-tubule, which induces the opening of the voltage-gated calcium channel from the sarcoplasmic reticulum. The released calcium ion binds to troponin C, which induces the cross bridge to contract. The resurgent Na^+^ currents introduce repetitive high-frequency firing, facilitating the binding leading to muscle stiffness. The essential tremors and chronic seizures found in the family members with a p.G1537S mutation in the *SCN4A* gene [29] may also have been attributable to the disturbance of the resurgent Na^+^ currents in the Na_v_1.4 channel.

In addition to SCM, mutations in Na_v_1.4 are also responsible for PMC. Clinically, mutations involving the Arg1448 of the *SCN4A* gene are frequently found in patients with PMC [30]. The resurgent Na^+^ currents in the Na_v_1.4 channel were first described using rat DRG neurons transfected with Na_v_1.4r-R1448P. Jarecki et al. reported that the resurgent Na^+^ currents could be obtained and that the average relative amplitude was 4.8± 0.7% [18], much lower than our findings (~10% in the WT and approximately 15–20% in mutant channels, Figure 4C). Furthermore, the Arg1448 locates at DIV/S4, which is the outermost extracellular charged residue in the sodium channel voltage sensor that modulates the activation/inactivation coupling of the channel. The study showed that the Na_v_1.4r-R1448P channels slow the inactivation and generate resurgent Na^+^ currents, suggesting that the mutation slows the rate of open-channel fast inactivation and may be able to induce resurgent Na^+^ currents. In another study, large resurgent Na^+^ currents could be observed in nearly 62% of Na_v_1.8-null DRG neurons using Biolistic transfected with Na_v_1.6r during the repolarization stage; however, no resurgent Na^+^ currents were identified in neurons transfected with a TTX-R version of the rat skeletal muscle sodium channel Na_v_1.4, although they generated a larger peak sodium current amplitude than the Na_v_1.6 channels [27]. The discrepancy may have been due to the different expression systems and the modification of the channel to become TTX-resistant since resurgent Na^+^ currents are difficult to obtain using DRG neurons or WT skeletal muscles [27]. In a recent report, a p.G1306E mutation of SCN4A gene caused NDM and Brugada syndrome after treated with flecainide. The different isoforms of Nav1.4 expressed in the skeletal and cardiac muscles may result in such a rare phenotype [31].

In conclusion, mutations in *SCN4A* are responsible for SCM. The hyperpolarizing shifting of the activation and inactivation curves, which results in an increase of window and sustained Na^+^ currents, may contribute to the hyperexcitability of neural-muscle tissues. The Na_v_1.4 channel can generate resurgent Na^+^ currents, which may further increase repetitive firing of the action potential in skeletal muscle, one of the hallmarks of SCM in EMG. In addition to myotonia, patients with SCM frequently experience episodes of muscle weakness. Overall, *SCN4A* mutations resulting in defects of slow inactivation and delay of time to peak in resurgent Na^+^ currents are likely factors contributing to constant myotonia and weakness in SCM patients.

## 4. Materials and Methods

### 4.1. Genetic Analysis of Two Families with Non-Dystrophic Myotonia (NDM)

Two probands with NDM from two individual Taiwanese–Chinese families visited the neurology clinic of the National Taiwan University Hospital, Taipei, Taiwan. Leukocyte DNA from the probands and their relatives was collected for genetic diagnosis. The genetic studies were approved by the Institutional Review Board (NTUH-REC No.: 201802049RINB) and written inform consent was obtained. Genetic analysis of two families with non-dystrophic myotonia (NDM). All methods and procedures were performed in accordance with the guideline and regulations.

### 4.2. Preparation for DNA Constructs

The cDNA of the wild-type (WT) human Na_v_1.4 channel (*SCN4A*) was purchased from OriGene Technologies Company (Cat. No. RC218290; Rockville, MD, USA). For mutagenesis, p.V445M mutant channel cDNA was created using the QuikChange Site-Directed Mutagenesis System Kit (Stratagene, La Jolla, CA, USA), and a c.1333G>A sequence variant was introduced. The cDNA sequence was confirmed by automatic DNA sequencing (3730xl DNA Analyzer; Applied Biosystems, Foster, CA, USA).

### 4.3. CHO-K1 Cell Cultures for WT and p.V445M Mutant cDNA Transfection

The Chinese hamster ovary (CHO-K1) cells used were approved by the Food Industry Research and Development Institute, Hsinchu, Taiwan. The cells were maintained in F12-K medium (Thermo Fisher Scientific, Waltham, MA, USA) under humidified conditions with 5% CO_2_|95% O_2_ at 37 °C. The medium was supplemented with 10% fetal bovine serum (Thermo Fisher Scientific) and 0.5% penicillin-streptomycin antibiotics (Thermo Fisher Scientific). A total of 1 × 10^6^ CHO-K1 cells were seeded into a 35-mm culture dish (Greenpia Technology, Seoul, Korea). Briefly, Lipofectamine™ 3000 (Thermo Fisher Scientific, USA) DNA mix with 5.0 μg WT or p.V445M *SCN4A* cDNA construct and 0.1 μg green fluorescent protein was added to CHO-K1 cells in the F12-K medium at 37 °C with 5% CO_2_|95% O_2_ for one to two days. For the whole-cell patch-clamp recording, 0.1 mg/mL trypsin (Sigma-Aldrich, St. Louis, MO, USA) was added to the transfected cDNA CHO-K1 cells and let the cells to be dissociated in F12–K medium with 10% FBS. Then, the cells were plated on coverslips under 37 °C for ~60 min. Usually the patch-clamp recordings were carried out within 2–3 days following cDNA transfection, and the isolated single cells for electrophysiology recording were used within ~4 h of preparation.

### 4.4. Whole-cell Patch-Clamp Recordings of CHO-K1 Cells Expressing WT and p.V445M Mutant SCN4A Constructs

Whole-cell patch-clamp recordings of CHO-K1 cells expressing WT and p.V445M mutant *SCN4A* cDNA constructs were performed two to three days after the transfection. The recordings were carried out using an Axopatch 700B amplifier (Axon Instruments, Sunnyvale, CA, USA) and the pClamp 9.2 acquisition software package (Molecular Devices, San Jose, CA, USA). The currents were digitized with the Digidata-1322A interface (Axon Instruments, USA). A glass electrode pipette was fabricated with a tip of approximate diameter 1.0 μm and fired to a resistance of 1.0–2.0 mΩ using the Sutter P-97 puller (Sutter Instrument Company, USA). The glass electrode pipette was filled with an internal solution containing 75 mM CsCl, 75 mM CsF, 5 mM HEPES, 2 mM CaCl_2_ and 2.5 mM EGTA (pH = 7.4 – 7.6). The whole-cell configuration was immersed in an external solution of 145 mM NaCl, 10 mM HEPES, 2 mM CaCl_2_ and 2.5 mM MgCl_2_ (pH = 7.4 – 7.6). Then, the whole-cell configuration was moved in front of square glass barrel micropipes (Drummond Scientific, Broomall, PA, USA) emitting the extracellular solutions. A short intracellular Na_v_β4 peptide (KKLITFILKKTREK−OH, 0.1 mM) was also added to the internal solution to generate resurgent Na^+^ currents. One micromolar of tetrodotoxin (TTX) (Tocris, Bristol, UK) was used to block TTX-sensitive (TTX-s) Na^+^ channels. By subtracting the TTX-s currents, the resurgent Na^+^ currents could be obtained. To estimate the conductance, the data were fitted into the following Boltzmann sigmoid equation: Y = 1/1 + exp[(V_h_ − X)/*k*], where Y is the conductance, X is the membrane potential, V_h_ is the membrane potential with half of the conductance, and *k* is the slope of the curve.

### 4.5. Homology Modeling

The homology modeling procedure was performed using methods similar to those used in our previous study [13,21]. A homology model of the WT Na_v_1.4 channel encoded by the *SCN4A* gene was built from X-ray crystal structure data of the human voltage-gated Na_v_1.4 channel (human Na_v_1.4; PDB code: 6AGF). The amino acid sequence of the WT Na_v_1.4 channel was obtained from the UniProt database (P35499). Sequences of the three inter-domain linkers of the WT Na_v_1.4 channel (DI–DII linker, A447-P578; DII–DIII linker, L798–W1032; and DIII–DIV linker, G1292-A1354) were inserted into positions between the domains (DI–DIV) via homology modeling based on the WT human Na_v_1.4 channel. The aligned sequences were then processed using Discovery Studio 2018 (Dassault Systèmes, Vélizy–Villacoublay, France) to assign the relative positions and generate the secondary structure of the WT human Na_v_1.4 channel [13,21].

### 4.6. Data Analysis

All statistical data were described as mean ± standard error mean. Statistical significance was assessed and analyzed using the Student’s independent *t*-test and accepted at *p* < 0.05.

## Supplementary Materials

Supplementary materials can be found at https://www.mdpi.com/1422-0067/21/7/2593/s1.
