# Supplementary material for: Changes of Resurgent Na+ Currents in the Nav1.4 Channel Resulting from an SCN4A Mutation Contributing to Sodium Channel Myotonia
Source: Int J Mol Sci. 2020 Apr 8;21(7):2593. doi: 10.3390/ijms21072593 (PMC7177622; doi:10.3390/ijms21072593)
Supplement: Supplementary file 1 [file ijms-21-02593-s001.pdf]

Supplementary materials

Article

Changes of resurgent Na^+ currents in the $Na_v1.4$ channel resulting from an *SCN4A* mutation contributing to sodium channel myotonia

Chiung-Wei Huang,[1] Hsing-Jung Lai,[2,3,] Pi-Chen Lin,[4] Ming-Jen Lee[2, 5 *]

1. Institute of Physiology, Kaohsiung Medical University, Kaohsiung, Taiwan; g10054b@ms51.hinet.net
2. Department of Neurology, National Taiwan University Hospital, Taipei, Taiwan; mjlee@ntu.edu.tw
3. Department of Neurology, National Taiwan University Hospital Jinshan Branch, New Taipei City, Taiwan; i5492111@gmail.com
4. Division of Endocrinology and Metabolism, Department of Internal Medicine, Kaohsiung Medical University Hospital, Kaohsiung Medical University, Kaohsiung, Taiwan; pichli@kmu.edu.tw
5. Department of Neurology, National Taiwan University Hospital Yunlin Branch, Yunlin, Taiwan; mjlee@ntu.edu.tw

* Correspondence: mjlee@ntu.edu.tw; Tel.: +886 2 23123456 ext 65342

Figure S1

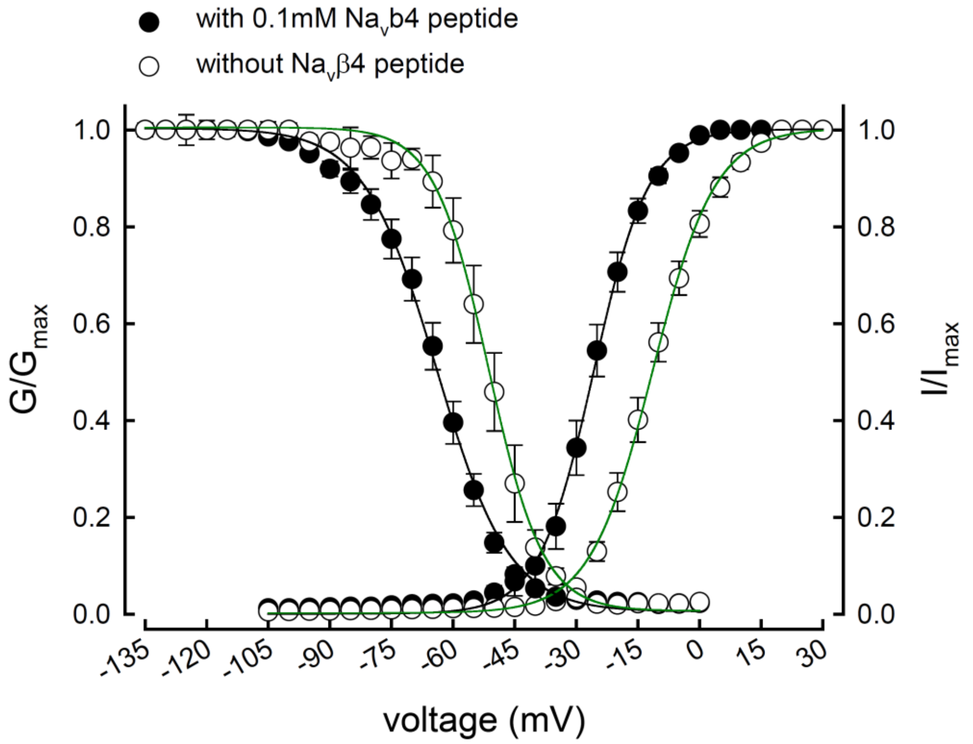

Figure S1. The activation and inactivation curves of the WT $Na_v1.4$ channel with and without of the 0.1mM $Na_v\beta4$ peptide

The activation and inactivation curves for each cells are fitted with Boltzmann functions, and the cumulative results for V_h are –25.79±1.5 mV and –11.43±1.21 mV with and without the 0.1 mM $Na_v\beta4$ peptides, respectively (n= 9; $p<0.05$), and *k* are 6.67±0.43 and 7.59±0.34 with and without the 0.1 mM $Na_v\beta4$ peptides fort the inactivation curve, respectively (n= 9; no significant difference). For the inactivation curves, the V_h are –63.99±2.17 mV and –51.35 ±1.51 mV with and without the 0.1 mM $Na_v\beta4$ peptides, respectively (n= 9; $p<0.05$), and *k* are –8.93±0.33 and –6.62±0.38 with and without the $Na_v\beta4$ peptides, respectively (n= 9; no significant difference).

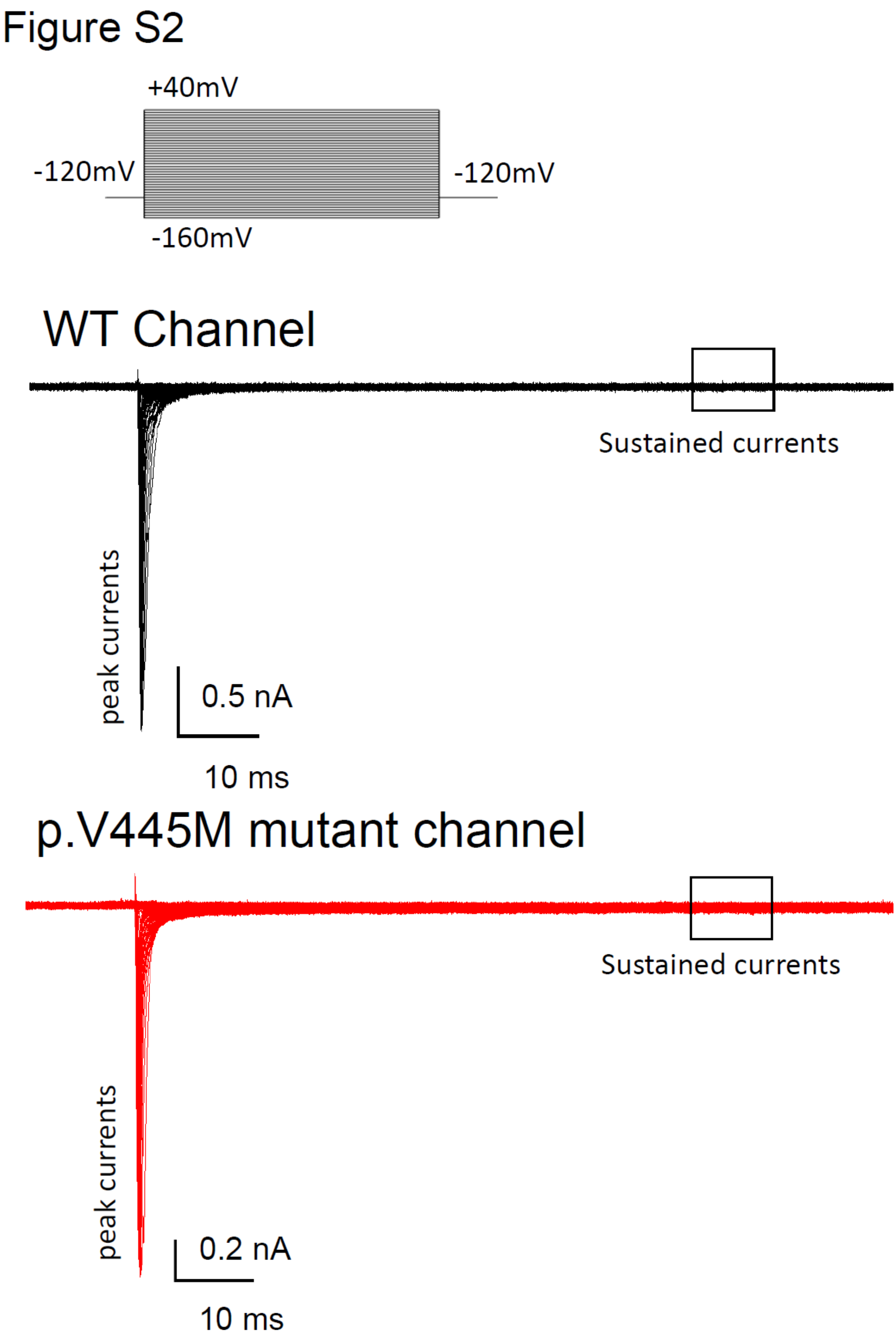

Figure S2. Sample sweeps shows the peak currents and sustained currents in WT and p.V445M mutant Na$_V$1.4 channels

Sample sweeps traces recorded from CHO-K1 cells expressing WT $Na_v1.4$ and p.V445M mutant channels in responded to activation stimulation protocol (~100 ms pulse). Sustained currents are seen as current remaining at the end of the 90-95 ms of stimulus duration. Note that the sustained currents are larger in the p.V445M mutant $Na_v1.4$ channel compared to the WT channel.
